# Management of The Elderly Cancer Patients Complexity: The Radiation Oncology Potential

**DOI:** 10.14336/AD.2019.0616

**Published:** 2019-06-16

**Authors:** Giuseppe Colloca, Luca Tagliaferri, Beatrice Di Capua, Maria Antonietta Gambacorta, Vito Lanzotti, Andrea Bellieni, Silvio Monfardini, Lodovico Balducci, Roberto Bernabei, William C Cho, Vincenzo Valentini

**Affiliations:** ^1^Fondazione Policlinico Universitario Agostino Gemelli IRCCS, UOC di Radioterapia, Dipartimento di Scienze Radiologiche, Radioterapiche ed Ematologiche, Roma, Italy.; ^2^Istituto di Medicina Interna e Geriatria, Università Cattolica Sacro Cuore, Roma, Italy.; ^3^GIOGER Gruppo italiano di Oncologia Geriatrica, Italy.; ^4^Geriatric Oncology Program Istituto Palazzolo, Milano, Italy.; ^5^Senior Adult Oncology Program Moffitt Cancer CenterTampa, USA; ^6^Department of Clinical Oncology, Queen Elizabeth Hospital, Hong Kong.

**Keywords:** radiation oncology, elderly, cancer, frailty, quality of life, sarcopenia, complexity, personalized treatment

## Abstract

Radiation oncology has the potential to be an excellent option for the frail elderly cancer patients because of its limited systemic toxicities. It can be effective for curative, prophylactic, disease control or palliative purposes. Currently about 60% of all cancer patients undergoing active treatment at some point receive radiation treatment. However, though widely used, there are limited clinical trials strictly designed for the elderly. This paper will review the key points in the assessment and treatment of elderly cancer patient including quality of life, active life expectancy, cognitive performance, frailty, sarcopenia and how the new technologies can help to reach the key goal of maintaining autonomy and independence for the elderly cancer patient.

With aging population and with life expectancy reaching 82 years old for women and 75 years old for men in the Western world, it is not surprising that cancer will be an older adults’ disease. Furthermore, by 2030, it is projected that more than 70% of new cancer diagnoses will be in the elderly [1].

Moreover, elderly patients aren’t frequently offered appropriate cancer therapies because of their age and because so often the physicians do not have the proper skills to assess the complexity of elderly patient or to recognize the functional limits that frail elderly individuals have.

There is general agreement of the fact that age should not be the deciding factor for the elderly who are seeking cancer treatments. Conversely, physical and cognitive performance, multimorbidities, patient will, compliance and the cloud of emotions surrounding the patient after a cancer diagnosis should be taken more into consideration within the process of treatment decision.

Radiation oncology is a cancer management approach that can be an excellent option for the frail elderly because of its limited systemic toxicities. It can be effective for curative, prophylactic, disease control or palliative purposes. Currently about 60% of all cancer patients receiving active treatment at some point have radiation as part of their therapeutic strategy, but although widely used, there are limited clinical trials designed strictly for the elderly. Radiation oncology does have potential disadvantages for elderly frail population, for example the long duration of treatment, especially when the intent is curative and conventional fractionation is employed, site-related toxicities that may be more intense in the older adult. All this can affect quality of life and increase the need for additional medical, surgical interventions or institutionalization. It is important to be aware of the acute symptoms that can be prodromic to chronic clinical issues in the ongoing care of the elderly after radiation therapy (for example whole brain irradiation- cognitive impairment, pelvis - marrow aplasia or radiation enteritis) and therefore, it is crucial to distinguish between physiological aging changes [2] and radiation therapy's acute and long-term toxicities.

## The meaning of aging in the cancer patients

Aging is defined as a progressive functional decline, or a gradual deterioration of physiological function or the intrinsic, inevitable, increase in vulnerability [3,4].

It is characterized by several individual changes including loss of muscle and bone mass, a lower metabolic rate, longer reaction times, declines in cognitive functions, sexual activity, changes in organ and immune functions (immunosenescences), pain threshold, and in exercise performance [2,5,6].

This definition, useful in gerontology, makes no sense in front of a new cancer diagnosis. The need to start cancer treatment, surgery, the cancer itself, profoundly alter the patient's homeostasis so as to make the cancer itself as a kind of frailty stress test. In the clinic, when faced with an oncology patient, it becomes more important to consider his active life expectancy, rather than his biological or chronological age. Therefore, consider the therapeutic options based on the patient's life expectancy, on the quality of life perceived by the patient himself, herselg, on the physical and cognitive performance of the patient, rather than on the basis of biological or chronological age.

Before submitting the patient to any type of treatment or to make a management choice, it is important to consider the patient’s average number of years of life remaining in an independent state, free from significant disability. Let's try to imagine a 75-year-old patient, woman with no comorbidities, she has 15.3 years of life expectancy, while if she had a high comorbidity index the life expenctancy is 8.5 years. Try to imagine a therapeutic choice thinking not about the biological and chronological age of this patient, but in the years that could potentially face, could avoid both under-treatment and over-treatment [7]. An approach of this type translates into a radical change of vision. The geriatric skills associated with oncological technologies should therefore focus both on a target therapy and on the personalization of the treatment, understood as following the patient and supporting him throughout the course of treatment in order to maximize results and reduce toxicity. This is possible only by combining the new therapies, the best technologies and the geriatric mindset. With the objective in the geriatric assessment to evaluate all those aspects that can have a considerable impact on the treatments and at the same time is not correctly intercepted by oncologists, as the cognitive impairment, sarcopenia and frailty. Cognitive performance is probably the most important aspect in the oncological field to consider, as it subtly impacts both on the compliance to the treatments and on the toxicity related to the treatments themselves and above all on the premature interruption of the treatments.

Patients with cognitive impairment are more likely to suffer from delirium and reduction in cognitive performance after or during an oncological treatment. The long bed stays tied to delirium is a risk factor for infections, pressure ulcers, loss of ability or independence and terminal disease.

The failure to evaluate memory or cognitive compliance is likely to hinder treatment of underlying conditions of disease and comorbidity and may present safety issues for the patient. Many patients who are developing or have cognitive impairment do not receive a diagnosis and more than half of patients with dementia had not received a clinical cognitive evaluation by a physician [8]. This is not unusual if we think about how physicians were unaware of cognitive impairment in more than 40 percent of their cognitively impaired patients [9].

Cognitive impairment in older adults has a variety of possible causes, including medication side effects, metabolic and/or endocrine imbalance depression, dementia, and delirium due to undercurrent illness.

Clinicians should remain alert to early signs or symptoms of cognitive impairment (for example, issues with memory or speech) [10].

Some causes, like medication side effects and depression, can be reversible, others, such as dementia, cannot be regressed, but symptoms can be treated for a period of time and families can be prepared for predictable changes, and to balance the possible alterations / deteriorations that may occur due to the treatments with the advantages of the oncological treatments themselves [11].

While screening tests are insufficient alone, crucial is the first step to understand if cancer patients need a deeper geriatric evaluation as like a Geriatric Assessment (GA)[12].

**Figure 1.**
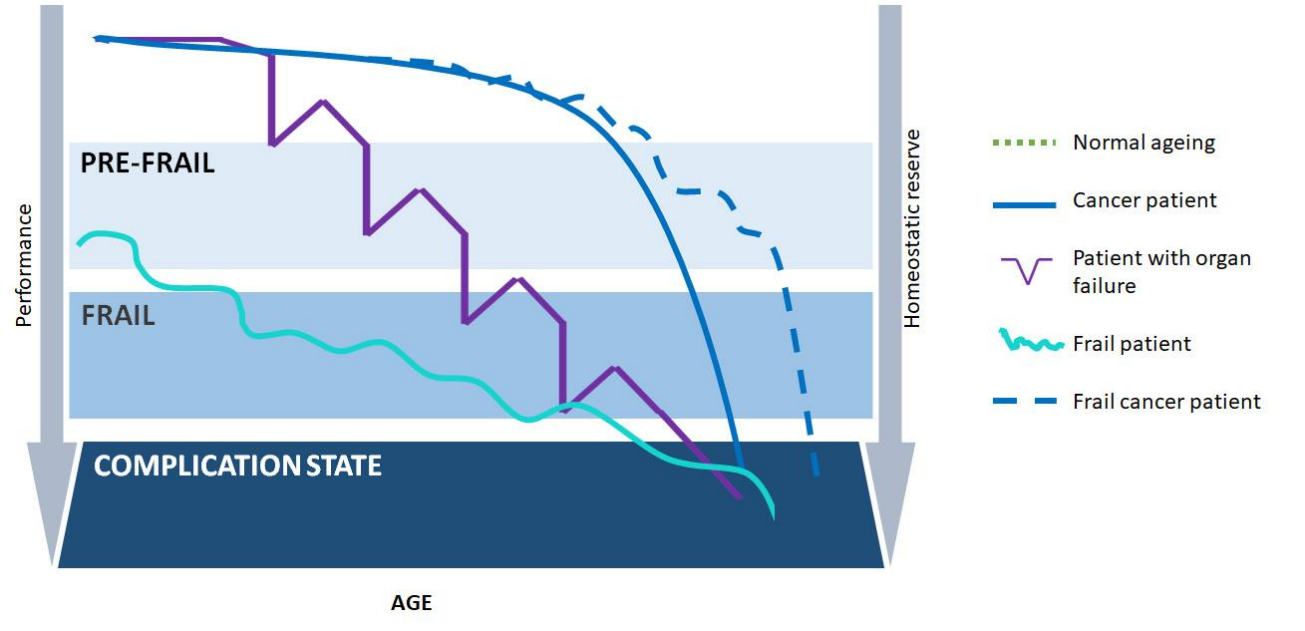
Comparison between successful aging, organ failure, fragility, cancer.

### The Frailty and complexity scenario in the elderly cancer person

Frailty was described as "a state of increased vulnerability to stressors due to age-related declines in physiologic reserve across neuromuscular, metabolic, and immune systems" [13]. This definition of frailty has evolved over the years from a description of dependence on others to a more dynamic model that encompasses biomedical and psychosocial aspects. Frailty is an extended process of increasing vulnerability to minor stressors and risk for adverse outcomes [14], predisposing to functional decline and ultimately leading to death.

Frail has to be considered not a synonym for comorbidity or disability, but it is an interaction of a person’s assets and deficits as a result of the combination of a series of factors such as age, gender, lifestyle, socioeconomic background, comorbidities, affective, cognitive or sensory impairments

In this scenario, frail individuals are less able to adapt to stressors such as acute illness, surgical intervention or new medication, with an increased risk for multiple adverse outcomes, including institutionalization, disability and death. on these basis cancer should be considered as a sort of frailty stress test [15] (Table 1).

On the other hand, if we try to compare with the curve of successful aging, the curves of organ failure, that of frailty and that of cancer, we can see that thanks to the new oncological treatments, cancer chronicization is bringing this curve to take on more and more the characteristics of that of frailty (Fig. 1).

If an elderly patient develops cancer, does he become frail? At the same time how to assess a frail patient who develops a cancer? Can a fit patient become frail during cancer treatment?

The attempt to answer these questions represents the complexity scenario in front of which we find ourselves when we decide to treat an elderly cancer patient.

Multiple frailty screening tools have been developed and utilized for risk assessment and epidemiologic study. The majority of them have been developed based on the comparison between two concepts: "physical frailty” versus "deficit accumulation frailty”[16].

The physical approach conceptualizes frailty as a syndrome that is driven by altered stress response systems and age-related molecular changes, resulting in vulnerability to morbidity and mortality. The deficit accumulation approach conceptualizes frailty as a cumulative burden of physical and psychological illness, disability and social factors that puts an individual at an increased risk for additional adverse outcomes. This approach combines tallies of medical, physiologic, cognitive, and social factors to identify frailty status [17,18].

None of these definitions can cover the needs of assessemnt of an elderly cancer patient, predict the response to a treatment or reduce the risk of toxicity related to a treatment. Absolutely none of these definitions is able to accurately describe the scenario in which the character represented by the oncological elder is recited

There is no gold standard for elderly patients with cancer thus the ability to establish a diagnosis of frailty and to provide effective intervention to prevent progression to disability remains the major challenges in managing the patient’s complexity [12]. Probably, Sarcopenia condition that has been widely associated to frailty and it has been proposed as the underlyng condition of frailty itself becouse strictly related to negative outcomes as toxicities and mortality could become the keystone in the assessment of the elderly cancer patient [19].

**Table 1:** Instrument used to perform geriatric assessment in oncological patients.

Domain	Tool
Social status	MOS Social Activity Survey Caregiver burden
Multimorbidity	Charlson Comorbidity Index CIRS CIRS-G Number of comorbid conditions Simplified Comorbidity Score Summary of comorbidities
Functional status	ADL: Katz index IADL: Lawton scale Performance status index Barthel Index Pepper Assessment Tool for Disability Visual and/or hearing impairment, regardless of use of glasses or hearing aids MOS physical Health (any version) Mobility Problem (requiring help or the use of a walking aid) Timed Get Up and Go Hand grip strength Short Physical Performance Battery One-leg standing balance test Gait speed
Cognition	MMSE (any version) Montreal Cognitive Assessment (MoCA) Informant Questionnaire on Cognitive Decline in the Elderly Clock-drawing test Blessed Orientation-Memory-Concentration Test
Depression	GDS Center for Epidemiologic Studies Depression Scale HADS Mental Health Index The distress thermometer
Nutrition	BMI MNA Short nutritional assessment questionnaire
Polypharmacy	Beers criteria STOP and START criteria

### The application of modern radiation oncology in the elderly

Technological advances in radiation oncology can provide a personalized treatment strategy in order to propose a curative or palliative option for elderly patients [20, 21].

Radiation therapy is a key treatment in the multidisciplinary management of cancer and can be proposed to older patients where frailty condition precludes surgery or upon completion of the surgery itself, without changing life expectancy, and maintaining a good balance between toxicity and results. In the absence of specific recommendations these patients tend to be undertreated because not deemed amenable for curative intent or over treated in the absence of a real benefit in survival with an increased risk of toxicity. Currently target of radiation therapy can be aged patients, even more than 90 years old, an extremely heterogeneous population in terms of health, functional and psychological status, social and economic wellness, with different health priorities.

There are four age-related factors to take in consideration when assessing the risk-benefit of a radiation treatment: 1- loco-regional tumor behavior that can be either more indolent or aggressive in older patients, 2- competing risks of non-cancer death and other morbid events that may change patient’s life expectancy, 3- functional reserve assessment, which is essential in the prediction of radiation-induced toxicity and patient’s ability to complete the prescribed radiotherapy treatment; 4- treatment goal, for example a curable setting may shift to palliation because patient’s life-expectancy and/or the functional reserve are compromised [22].

In the last 25 years radiation technique has shifted from a 2D planning to a 3D technique where the introduction of a computed tomography-based treatment planning has improved the treatment safety with anatomical characterization of target volume delineation and accurate dose calculation. The implementation of technologies such as intensity modulated radiation therapy (IMRT) and Image Guided brachytherapy (IGBT), with several radiation beams coming from different angles and a modulation of the intensity of the beam path and the shaping of each beam, allows to carve the dose on the tumor. These adjustments enable the prescribed amount of radiation to be delivered to each part of the tumor, while minimizing exposure to the surrounding healthy tissue. A growing number of evidence [23-26] has shown the superiority of IMRT in normal tissue sparing, in particular for bone marrow, small bowel, parotid gland, rectum, with a significant reduction in the rate, intensity and duration of radiation-induced enteritis [27], proctitis, pelvic fractures, xerostomia [28] and hematologic toxicity [24], to cite some. The consequence is a reduction in both acute and late toxicity, which may be beneficial especially in older patients. Volumetric modulated arc radiotherapy (VMAT) technique, with a faster delivery compared to static IMRT, allows the use of radiotherapy in elderly patient. VMAT is more comfortable for this kind of patients who experiment difficulties in standing still on a rigid plan or holding back a full bladder, for delivery protocols. The precision of these techniques has focused the attention on the reproducibility of daily treatments in order to avoid the so called “target missing”. This problem can be amplified in an older patient for constipation or bladder weakness that is often an issue and can directly influence organ and tumor motion. Moreover, the patient’s involuntary movement due to impaired mobility can impact on daily set up. In this context the growing use of image-guided radiation therapy (IGRT) allows the reduction of the daily set-up errors, through the use of on-board CT imaging system.

Another important issue in the elderly is the overall radiotherapy time. Long course treatments are time consuming and challenging in a frail patient and can represent a cause of poor compliance or even of early interruption of the treatment. The reason for decline includes the fear of side effects, the unclear benefit of treatment, the feeling of being too old for treatment and the desire of maintaining a good quality of life, as well as financial reasons, poor communication with physician, travelling needed for treatment, fear of loss of independency [29]. Patients need to be adequately informed in order to involve them in the decision of the optimal treatment.

In the context of an individualized treatment approach, physician should consider tumor-related and older patient-related characteristics, has to be aware of alternative fractionation schedule and technological advances that could reduce treatment time and toxicity while maintaining the same efficacy. This approach would be probably better accepted by the patient and would translate in a better treatment compliance and outcomes.

Stereotactic and hypofractionated radiotherapy can be considered a valid option to be offered to selected elderly patients, as reported in breast [30], lung [31], pancreatic [32], rectal [33] and central nervous system cancers [34] as in brain [35], liver or lung metastases [36,37]. Different hypofractionated schedules are now in use each demonstrating similar rate of local control and late cosmetic outcomes [30,38,39].

### New technologies that could be applied to the elderly

Over the past 20 years there were a significant improvement in the field of Interventional Radiotherapy (brachytherapy BT) that covered in particular the planning, delivery and surgical techniques with multidisciplinary approach [40]. Hence, modern BT determines an advantage especially in the management of relapses and in early stage disease than other treatment, [41] being a localized treatment, reducing the toxicity compared to external beam radiation and chemotherapy. BT used as a boost, is less invasive than surgery and leads to the use safer anesthetic techniques, especially for the elderly, such as local or spinal anesthesia or mild sedation. Brachytherapy may find increasing use in the intra/perioperative irradiation by endoscopy [42] and the use of hypofractionated treatment schemes. This allows a lower overall treatment time that offers radiobiological advantages but also a treatment more comfortable for the patient.

New technology, recently, implemented is the use of RM. It allows to improve the treatment because it is possible to have the MR information during the treatment, in fact it has been demonstrated that lesions moving with small amplitude show limited amplitude variability throughout treatment, making passive motion management strategies seem adequate. However, other variations such as baseline drifts and shifts still cause significant geometrical uncertainty, favouring real-time monitoring and an active approach for all lesions influenced by respiratory motion [43.44]. Technological advances in radiation oncology exploited successfully the use of different particles (heavy charged ions, like protons), because of their physical characteristic. The key point of proton therapy (in particular the pencil beam scanning Intensity Modulated Proton Therapy delivery technique is the superior dose distribution, allowing to precisely aim the highest dose at the tumor and avoid healthy tissues. Consequently, it shows many advantages over x-ray therapy [45]. Obviously, other several clinical indications for adults and elderly take advantages of proton therapy and several long-term outcomes [46] are finally getting available from the always most increasing number of worldwide centers. Thanks also to the always increasing level of technology, both in delivery both in the patient in-treatment verification, an increasing number of new fractionation scheme and dose level is under evaluation in several clinical trials or already in clinical operation [45,46].

### Back to the future: fostering patient autonomy via mobile app for a better patient empowerment

The healthcare is undergoing an evolutionary phase worldwide, with the need to face multiple challenges such as the aging of the global population, the unsustainable health care delivery costs and the trend toward a mobile society [49].

A growing body of research suggests that people actively involved in their healthcare tend to have better clinical outcomes - and may incur lower healthcare costs. Therefore, there is more and more interest in the medical community in developing and improving health services with the addition of the so-called patient-reported outcomes (PROs) to traditional clinical endpoints. Mobile engagement, among other strategies, is proving to be particularly successful in multiple areas, such as wellness and lifestyle management, medication adherence and patient education [50]. Mobile Health (mHealth) is a broad concept including various types of mobile technologies. It often refers to consumer health care technologies, such as Web-based information resources, telephone messaging (short message service/SMS, multimedia messaging service/MMS), remote monitoring of patients, remote interpretation of medical reports, videoconferencing, and telehealth, including the remote services of a surgeon operating at a distance, and telerobotics [51]. More in details, the World Health Organization has underlined that mHealth includes several technologies like mobile phones, personal digital assistants (PDAs), and smartphones, patient monitoring devices, mobile telemedicine/telecare devices, MP3 players for mLearning, and mobile computing.

However, with more than 40,000 Medical Health Apps available today, finding a truly efficient patient engagement app is a real challenge. Several apps do little more than providing information; such information is often unreliable, as only two thirds of mHealth apps are actually used after being downloaded; few are used more than once, and fewer still demonstrate measurable results [52] .

A customized, condition-specific outpatient care app may help to ensure that recently discharged or outpatient customers stay informed, engaged and connected throughout their healthcare journey. A health comprehensive cancer care app helps improving outpatient experience by allowing patients to:

- Track their treatment, medications & lab results;
- Build and manage their care team
- Track their daily pain levels or feelings
- Network and socialize with other patients

In radiotherapy these tool will become essential to follow the patient during and after the treatment; patient can report his/her symptoms, like pain or other discomfort, that can be treated early preventing severe toxicity and discontinuation of the treatment; the app could also track symptoms or lab parameters that can represent the first warning of a severe complication of the therapy. Finally, we can check if our patient is maintaining a healthy and active lifestyle, that is foundamental in elderly population. Outpatient care apps can have tremendous impact on patient care costs by reducing preventable readmissions and helping patients adhering to medications. They can also serve as excellent patient education tools and the basis for large database [53,54].

**Table 2:** Key points to assess before decision making in elderly cancer patients.

Phisical performance (is he/she fit, frail, pre-frail?)
Cognitive performance (needs for cognitive assessment?)
Active life expenctancy
Quality of life (individual needs and will)
The enviroment (the social cloud around the patient is able to allow the treatment?)

### Conclusion

**Figure 2.**
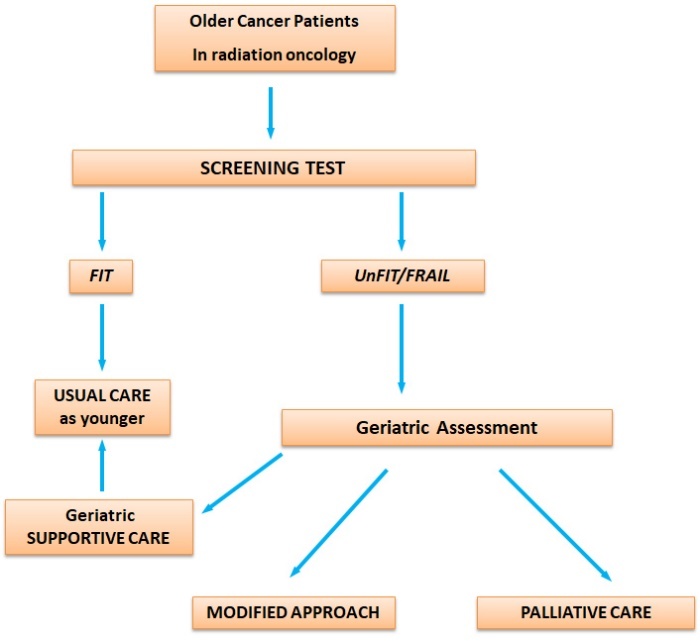
Flow Chart: Decision making in radiation oncology on elderly cancer patients. For supportive care we mean those cares focuse on preventing and treating symptoms or any complication due to cancer or therapies, during oncological treatment. For palliative care we mean a multidisciplinary approach dedicated to patients with life-threatening illness no longer treatable for cancer, that prevent and relief physical, psychosocial and spiritual sufferings.

With the aging population, the approach to older cancer patients needs to change. Comorbidities or aging should not be regarded upfront as contraindications to the optimal treatment. The older patient should have the same access to modern radiotherapy as his younger counterpart. The new technologies applied to the elderly can make it possible to obtain excellent results in terms of cancer control and symptom management with less toxicity and maintaining a good quality of life. This scenario certainly implies a specific approach in collaboration between radiotherapists, oncologists and geriatricians (Table. 2) [55]. Especially geriatricians must begin to learn about the new technologies available to help oncologists and radiotherapists in the better management of elderly cancer patients, to focus on maintaining autonomy and independence for older people, assessing new parameters as quality of life, active life expectancy, cognitive and physical performance and considering new technologies that can support elderly cancer patients in the healthcare process (Fig. 2).
